# Bacterial Gut Symbionts Contribute to Seed Digestion in an Omnivorous Beetle

**DOI:** 10.1371/journal.pone.0010831

**Published:** 2010-05-26

**Authors:** Jonathan G. Lundgren, R. Michael Lehman

**Affiliations:** North Central Agricultural Research Laboratory, United States Department of Agriculture–Agricultural Research Service, Brookings, South Dakota, United States of America; Agroscope Reckenholz-Tänikon, Research Station ART, Switzerland

## Abstract

**Background:**

Obligate bacterial symbionts alter the diets of host animals in numerous ways, but the ecological roles of facultative bacterial residents that colonize insect guts remain unclear. Carabid beetles are a common group of beneficial insects appreciated for their ability to consume insect prey and seeds, but the contributions of microbes to diet diversification in this and similar groups of facultative granivores are largely unknown.

**Methodology and Principal Findings:**

Using 16S rRNA gene clone libraries and terminal restriction fragment (tRF) length polymorphism analyses of these genes, we examined the bacterial communities within the guts of facultatively granivorous, adult *Harpalus pensylvanicus* (Carabidae), fed one of five dietary treatments: 1) an untreated Field population, 2) Seeds with antibiotics (seeds were from *Chenopodium album*), 3) Seeds without antibiotics, 4) Prey with antibiotics (prey were *Acheta domesticus* eggs), and 5) Prey without antibiotics. The number of seeds and prey consumed by each beetle were recorded following treatment. *Harpalus pensylvanicus* possessed a fairly simple gut community of approximately 3-4 bacterial operational taxonomic units (OTU) per beetle that were affiliated with the Gammaproteobacteria, Bacilli, Alphaproteobacteria, and Mollicutes. Bacterial communities of the host varied among the diet and antibiotic treatments. The field population and beetles fed seeds without antibiotics had the closest matching bacterial communities, and the communities in the beetles fed antibiotics were more closely related to each other than to those of the beetles that did not receive antibiotics. Antibiotics reduced and altered the bacterial communities found in the beetle guts. Moreover, beetles fed antibiotics ate fewer seeds, and those beetles that harbored the bacterium *Enterococcus faecalis* consumed more seeds on average than those lacking this symbiont.

**Conclusions/Significance:**

We conclude that the relationships between the bacterium *E. faecalis* and this factultative granivore's ability to consume seeds merit further investigation, and that facultative associations with symbiotic bacteria have important implications for the nutritional ecology of their hosts.

## Introduction

Microbes affect the phenotypes of their symbiotic hosts in myriad ways, especially the host's ability to rely nutritionally on certain foods. Nutritional symbioses between microorganisms and animals evolve when a major component of the animal's diet lacks sufficient quantities of specific nutrients, or when nutrients present in the diet are inaccessible because the animal lacks the requisite metabolic tools to fully digest their food [1], [2], [3], [4], [5]. Most research on nutritional symbioses has focused on how obligate relationships between microbes and their animal hosts evolve and are maintained [4], [6], [7]. Less understood are the functions of more transient or facultative bacterial communities that invariably reside within animal guts, which could contribute to the diet diversification of the host [1], [2], [3], [8].

Microbial-based nutritional symbioses are particularly well studied in insects with highly restricted diets of limited nutrition (e.g., blood, plant sap, wood, etc.) [4]. In these systems, bacteria or fungi help in nitrogen processing, sulfate assimilation, fatty acid metabolism, and help to contribute deficient sterols, vitamins (especially B-vitamin groups), digestive enzymes and essential amino acids to their insect hosts [8], [9], [10], [11], [12], [13], [14], [15], [16], [17], [18], [19]. Insects that feed on high quality foods (i.e., predators) or that display dietary plasticity (i.e., omnivores) were once believed to rely less on microbial symbionts, because these insects are able to self-select nutritionally optimal diets from their environment [20]. But even those insects that ordinarily consume diets of high nutritional quality often must ingest foods of marginal quality, either because high quality foods are temporally or spatially scarce or because “low quality foods” are superior in certain nutrients. The result of this is that most insects are best described as omnivores [5], [21], [22], and they must confront the physiological and structural hurdles associated with occasionally consuming certain suboptimal foods to attain a balanced diet [2], [3], [8]. Microbial symbioses are known to play a role in facilitating this omnivory in a number of insects (e.g., cockroaches, crickets, carpenter ants) [9], [10], [18], [23], [24].

Carabid beetles (Coleoptera: Carabidae) are a pervasive group of beneficial insects best appreciated for their usefulness as bioindicators of habitat qualities and for their contributions as predators of insect pests [25], [26], [27], [28], [29]. Additionally, several taxonomic clades of carabid beetles (especially within the tribes Zabrini and Harpalini) are important post-dispersal granivores [30], [31], [32], [33], [34] that help to regulate the dispersion and relative abundance of plant communities within agricultural and natural landscapes [5], [35], [36], [37], [38], [39]. The morphological bases that facilitate seed consumption by facultatively granivorous carabids are fairly well studied [5], [40]. However, although seeds are a highly nutritious food source, they are nutritionally and structurally distinct from the Carabidae's ancestral diet of arthropod prey [5], [41], [42], and the question remains how this dietary expansion evolved in this and similar insect groups.

Given the importance of microbial symbioses to the digestion of plant-based foods in other omnivorous insects [2], [17], we hypothesized that the gut bacteria of facultatively granivorous carabids contributes to their ability to digest seeds. Two recent studies [43], [44] have revealed a taxonomically simple community of gut bacteria within the digestive tract of three carabid species. Although these bacteria are apparently facultative symbionts (there are no species ubiquitously present within a population of beetles), they are autocthonous and nearly all bacteria discovered were representative of taxa that frequently live in association with higher organisms. Moreover, specific 16S rRNA gene sequences were frequently most similar to those recovered from other insect guts (i.e., they were not simply soil-dwelling species incidentally found in the beetle guts). The current research applies 16S rRNA gene clone libraries and terminal restriction fragment (tRF) length polymorphism analyses of five treatments to address whether, 1) alterations in diet affects the bacterial community of an omnivorous carabid beetle (*Harpalus pensylvanicus* [DeGeer]), 2) antibiotics reduce the bacterial community within guts of an omnivorous insect, and 3) reductions in specific bacterial taxa are associated with the capacity of a granivorous carabid to consume seeds.

## Materials and Methods

### Study organisms and feeding assays

All animals were handled in strict accordance with good animal practice as defined by the relevant national and/or local animal welfare bodies, and all animal work was approved by the appropriate committee. Beetles (n = 80) were collected nocturnally on 15-August, 2006 in Brookings, SD, USA (latitude, longitude: 44.340°, 96.790°). An additional population (n = 10 beetles) were frozen immediately after collection to allow comparison of the gut bacterial communities present in the laboratory-reared populations with those of field populations [44]. Beetles were provided with only a water-soaked cotton wick for 24 hr prior to the assay in individual, sterile, plastic Petri dishes (Falcon®, Becton Dickinson, Franklin Lakes, NJ, USA). The beetles were divided evenly and randomly into two dietary treatments, those fed eggs of *Acheta domesticus* (L.) (Orthoptera: Gryllidae) and those fed seeds of *Chenopodium album* L. (Amaranthaceae), a preferred seed species for this beetle [45]. Each beetle was offered 100 *A. domesticus* eggs or 175 *C. album* seeds for 24 hr. The numbers of food items consumed by each beetle were recorded as measure of pretreatment variability in consumption rates.

The beetle cohorts assigned to the prey and seed treatments were randomly divided equally between two subtreatments, those fed diet with antibiotics (termed aposymbiotic hereafter) and those fed unaltered diet (termed symbiotic hereafter). Specifically, following their initial feeding on eggs or seeds, aposymbiotic and symbiotic beetles were created by feeding them artificial diet [46] that contained tetracycline, rifampicin, and sorbic acid (0.04% w/w) or untreated artificial diet (respectively) for 10 d. Beetles were given only water for 24 hr, and then fed *A. domesticus* eggs or *C. album* seeds, according to their initial diet treatment. Beetle guts (without Malpighian tubules) were aseptically dissected in a Ringer's saline solution (0.75 g NaCl, 0.35 g KCl, 0.28 g CaCl_2_ per liter, pH 7.4) and frozen at −20°C until they were processed. Sample sizes for this final assay for aposymbiotic prey-fed, symbiotic prey-fed, aposymbiotic seed-fed, and symbiotic seed-fed were 19, 17, 13, 16, respectively.

### DNA extraction

Frozen, excised whole intestines were thawed on ice, washed 3× in sterile phosphate-buffered saline (PBS: 1.18 g Na_2_HPO_4_, 0.223 g NaH_2_PO_4_⋅H_2_0, and 8.5 g NaCl per liter; pH 7.5) and macerated with a sterile polypropylene micropestle. DNA was extracted from each intestine using the BIO101 FastDNA SPIN kit (Qbiogene, Inc., Carlsbad, CA, USA) per manufacturer's instructions. Each set of DNA extractions were accompanied by a negative extraction control (no intestine) and results were screened on a 0.7% agarose gel (100 V, 25 min). Negative extraction controls were carried through subsequent PCR and tRFLP analyses.

### Bacterial cell enumerations

The aseptically dissected intestinal tracts from three Field-collected *H. pensylvanicus* were fixed in ethanol (70%) and held at −20°C for estimates of total bacterial cell counts. Each gut was washed in PBS (3×), macerated with a sterile micropestle, vortexed with 1 mL 0.1% sodium pyrophosphate, sonicated (45 s, 125 W, 47 KHz) on ice, and re-vortexed prior to serial dilution in PBS. Aliquots of the gut suspension were filtered under vacuum onto 0.2-µm pore-size, black, polycarbonate membrane filters with cellulose-acetate support filters [47]. Cells concentrated on filters were stained with DAPI (0.01%, 3 min), washed, dried, and mounted in immersion oil (Cargille FF, Cargille Laboratories, Cedar Grove, NJ, USA) under a glass coverslip. Total bacterial cells were enumerated under epifluorescent illumination using a Leica DM LB2 microscope equipped with a 100× objective, 100-W mercury bulb, and filter set for DAPI (Chroma #31000, Chroma Technology, Rockingham, VT, USA). A minimum of five fields and 200 cells were counted or 20 fields when 200 cells were not achieved. Counts were conducted in triplicate for each intestine and averaged.

### Terminally-labeled restriction fragment length polymorphism (tRFLP)

Nearly full-length 16S rRNA genes were PCR-amplified in triplicate from the purified DNA extracted from each gut using universal eubacterial primers 8F (5′-AGAGTTTGATCCTGGCTCAG-3′) labeled with 6-carboxyfluorescein (FAM) at the 5′ terminus and 1492R (5′-GGTTACCTTGTTACGACYT-3′) [48] for tRFLP analysis [49]. PCR reactions (50-µL) were composed of 0.4 mg/L BSA (Roche Diagnostics, Indianapolis, IN, USA), 1X PCR buffer (GoTaq, Promega, Madison, WI, USA), 2 mM MgCl_2_, 0.5 µM of each primer, 1.25 U Taq DNA polymerase (Promega GoTaq), 0.2 mM each dNTP (Promega), 1 µL template DNA (ca. 25 ng DNA), and molecular grade water (Promega). PCR amplification was performed in a T-Gradient thermal cycler (Biometra, Goettingen, Germany) using the following conditions: 95°C (2 min); 30 cycles of 95°C (1 min), 55°C (1 min), 72°C (1.5 min); and a final elongation at 72°C (5 min). PCR products were screened on 1.2% agarose gel (75 V, 45 minutes) for the expected size product along with a size ladder and positive (*E. coli* DNA) and negative (reagents only) controls. Triplicate PCR products from each gut were then combined, purified (Wizard PCR preps, Promega), and quantified by absorbance at 260 nm. Combined, FAM-labeled PCR products for each gut were then restricted in triplicate (350 ng product; 10U *Rsa*1 and 1X NEB1 buffer, New England Biolabs, Beverly, MA, USA; molecular grade water, Promega) at 37°C (180 min) and terminated at 75°C (20 min). The triplicate digests with positive and negative controls were analyzed by capillary electrophoresis using filter D and Mapmarker 1000 size standards on an ABI Prism 3100 (Applied Biosystems Inc., Foster, CA, USA) operated under ABI's recommended run parameters. The resulting electropherograms were analyzed with Genemarker 1.5 (SoftGenetics, State College, PA, USA) using the Local Southern method of size calling, a threshold of 40 relative fluorescent units (RFU), a fragment range of 64–910 bases, and a peak window of 2 bp. Consensus terminal restriction fragment (tRF) profiles for each gut sample were prepared from the triplicate profiles using presence/absence and majority criteria.

### 16S rRNA gene clone libraries

16S rRNA gene clone libraries were constructed for each of the five groups of beetles using pooled DNA (one µL from each beetle in the group). Near full-length (ca. 1450 bases) 16S rRNA gene sequences were amplified (five separate reactions) from the pooled DNA extracts using primers 8F (unlabeled) and 1492R under the conditions and with the controls described previously for tRFLP. PCR products from the five reactions (for each library) were combined, quantified (abs 260 nm), purified (Wizard PCR preps; Promega) and cloned into *E. coli* JM109 competent cells using the pGEM-T Easy Vector System II (Promega) per manufacturer's instructions. For each of the five libraries, 95 clones were randomly selected and their plasmids containing the insert were purified (Montage Miniprep_96_, Millipore). The inserts were sequenced using the eubacterial bacterial primer 8F on an Applied Biosystems 3730xl DNA Analyzer. These partial sequences were trimmed and aligned using the RDPII pipeline tools [50] and a distance matrix was exported to DOTUR [51] for dereplication of each library. Several representatives for each OTU_0.97_ (i.e., operational taxonomic units with sequence similarities to identified taxa greater than 97%) from each library were then sequenced with the eubacterial primers 8F, 530F (5′-GTGCCAGCMGCCGCGG-3′), and 1100F (5′-GCAACGAGCGCAACCC-3′). Nearly full-length sequences were edited and assembled within BioEdit 7.5 freeware (http://www.mbio.ncsu.edu/BioEdit/page2.html). Clone assignments for each OTU_0__97_ for each library were confirmed with a second round of dereplication analysis using DOTUR. Potentially chimeric sequences that were identified following screening with Chimera_Check ver. 2.7 (RDP8.1), Bellerophon, [52], and Mallard [53] were removed from further consideration. Unique, representative sequences for each OTU_0.97_ were compared with entries in the GenBank database using BLASTn [54] to determine the closest database match. Unique sequences were deposited in GenBank under the following accession numbers: GU815101-GU815135. Calculations of diversity indices, the Chao1 estimator and rarefaction curves for each clone library were performed using FastGroupII [55]. Clones representing each OTU_0.97_ for each library were analyzed using the tRFLP procedures described above with the threshold set at 100 RFU.

### Data analyses

The relatedness of the bacterial communities in the five dietary treatments was measured using a hierarchical tree cluster analysis on the proportion of individuals in each treatment possessing each bacterial tRF, where distances are Euclidean and complete linkages were used to determine relatedness [56]. Discriminant analysis on the complete presence/absence data for each tRF was used to describe which bacterial tRF were most descriptive of the different treatments. In this analysis, prior probabilities were computed proportionally to the sample sizes of the different treatments. Factors with Eigenvalues >1 were included in the subsequent interpretations.

The mean number of seeds or prey consumed (log transformed) pre-treatment with antibiotics was compared with t-tests to ensure that treatments were initially equivalent in their consumption rates. Post-treatment consumption of seeds or prey (log transformed) were compared between aposymbiotic and symbiotic beetles fed each diet using t-tests. The relationship between individual bacterial tRFs and the number of seeds consumed by each symbiotic beetle were compared using a stepwise GLM. Six bacterial tRFs were not found in the symbiotic seed-fed treatment and were omitted from the analyses. Those beetles that did not eat seeds in the pre-treatment assay were omitted from the analysis. The categorical presence or absence of each tRF was compared with the log number of seeds consumed for each beetle. A forward, stepwise model (probability to include or exclude of 0.15) was used to reduce the number of tRFs included in the resulting model.

## Results

### Bacterial community in *Harpalus pensylvanicus* guts

Assuming a fresh gut weight of 40 mg and a density of one, we found 2.43×10^8^±1.80×10^8^ bacteria per ml gut (mean ± SEM, n = 10). There were 18 tRF identified in at least one of the 75 beetles. Of these 18 tRF, the 10 tRF observed in the Field population were the most common across all the beetles and were detected in beetles from three or more of the five treatments. The remaining eight tRF were uncommon, appearing in less than 10% of beetles from one or two of the treatments. Following dereplication of the five clone libraries, between six and nine unique OTU_0.97_ were found to represent the 16S rRNA gene sequence diversity for each library (Table 1). tRF analysis of these 35 representative OTU_0.97_ (total for all five libaries) produced only 12 unique tRF that included all but one (tRF 535) of the ten most common tRF from the individual beetles. Only two tRF were associated with an OTU_0.97_ representative from any of the five libraries that were not observed during the tRF analysis of the individual beetles: tRF 479 (clones P(a)6 and S(a)7; low abundance Betaproteobacteria) and tRF 870 (clone S(a)4; a low abundance Alphaproteobacteria); these three clones occurred a total of four times in the aposymbiotic clone libraries. Because dereplication was necessarily conducted at the library level to produce representative sequences for each sampling unit [library], similarity (based on shared best sequence match) among representative OTU_0.97_ from the five libraries is provided in Table 2. Accordingly, there were 24 unique OTU_0.97_ representing the 16S rRNA gene sequence diversity across all five libraries.

**Table 1:** Bacterial OTUs in *Harpalus pensylvanicus* stomachs fed one of five dietary treatments, identified using sequence information from the clone libraries.

	Clone	Relative abundance	Class affiliation	Closest cultured match (GenBank accession #)	Similarity (approximately 1450 bases; %)	tRF (bases)	Other clones with identical sequences
Field population	F1	24	Alphaproteobacteria	*Wolbachia pipientis*, (AY833061)	87.5	440	
	F2	23	Gammaproteobacteria	*Serratia rubidaea,* (AJ233426)	97.1	885	
	F3	19	Gammaproteobacteria	Coxiellaceae sp. (AF327558)	99.1	486	S(s)4
	F4	11	Gammaproteobacteria	*Citrobacter freundii* (DQ444289)	97.5	425	
	F5	9	Mollicutes	*Spiroplasma montanense* (AY189307)	85.2	472	S(s)5, S(a)2,
	F6	3	Gammaproteobacteria	*Proteus mirabilis* (AF008582)	97.6	425	S(s)7
Seeds (symbiotic)	S(s)1	42	Gammaproteobacteria	*Enterobacter hormaechei* (AJ853890)	99.1	425	
	S(s)2	6	Alphaproteobacteria	*Ehrlichia shimanesis* (AB074459)	87.7	440	S(a)3
	S(s)3	5	Bacilli	*Enterococcus faecalis* RO90 (AF515223)	99.3	903	P(s)4, P(s)8
	S(s)4	4	Gammaproteobacteria	Coxiellaceae sp. (AF327558)	98.1	486	F1
	S(s)5	3	Mollicutes	*Spiroplasma montanense* (AY189307)	87.1	472	F5 S(a)2,
	S(s)6	1	Gammaproteobacteria	*Acinetobacter calcoaceticus* (AJ888984)	96.6	885	
	S(s)7	1	Gammaproteobacteria	*Proteus mirabilis* (AF008582)	99.6	425	F6
Seeds (aposymbiotic)	S(a)1	42	Cyanobacteria (phylum)	none	NA	597	
	S(a)2	41	Mollicutes	*Spiroplasma montanense* (AY189307)	88.2	472	F5, S(s)5
	S(a)3	5	Alphaproteobacteria	*Ehrlichia shimanesis* (AB074459)	88.1	440	S(s)2
	S(a)4	2	Alphaproteobacteria	*Caedibacter caryophilus* BGD19 (AJ238683)	79.1	870	
	S(a)5	1	Gammaproteobacteria	*Serratia marcescens* (EU302855)	99.5	885	
	S(a)6	1	Mollicutes	Spiroplasma sp. “GENT” (AY569829)	99.4	812	P(s)9
	S(a)7	1	Betaproteobacteria	*Ralstonia pickettii* strain TA (DQ908951)	99.5	479	
Prey (symbiotic)	P(s)1	37	Gammaproteobacteria	*Pantoea dispersa* UQ68J (AY227805)	96.2	421	P(a)1
	P(s)2	13	Gammaproteobacteria	*Enterobacter aerogenes* (AJ251468)	97.4	421	
	P(s)3	12	Bacilli	*Lactococcus garvieae* M79 (AY699289)	98.8	896	P(a)3
	P(s)4	8	Bacilli	*Enterococcus faecalis* RO90 (AF515223)	99.1	903	S(s)3, P(s)8
	P(s)5	5	Gammaproteobacteria	*Proteus mirablis* I4320 (AM942759)	99.3	425	
	P(s)6	5	Gammaproteobacteria	*Serratia fonticola* (AY236502)	96.9	885	
	P(s)7	3	Gammaproteobacteria	*Citrobacter amalonaticus* (AF025370)	96.3	425	
	P(s)8	2	Bacilli	*Enterococcus faecalis* RO90 (AF515223)	93.9	903	S(s)3, P(s)4
	P(s)9	2	Mollicutes	Spiroplasma sp. “GENT” (AY569829)	99.2	812	S(a)6
Prey (aposymbiotic)	P(a)1	47	Gammaproteobacteria	*Pantoea dispersa* UQ68J (AY227805)	97.9	421	P(s)1
	P(a)2	17	Gammaproteobacteria	Acinetobacter sp. Dui-5 (EF031061)	99.4	425	
	P(a)3	13	Bacilli	*Lactococcus garvieae* M79 (AY699289)	99.0	896	P(s)3
	P(a)4	8	Gammaproteobacteria	*Serratia marcescens* (EF208031)	99.7	885	
	P(a)5	2	Betaproteobacteria	*Delftia acidovorans* SPH-1 (CP000884)	99.1	425	
	P(a)6	1	Betaproteobacteria	*Ralstonia pickettii* 12j (CP001069)	96.4	479	

**Table 2:** Diversity indices for the 16S rRNA gene clone libraries using OTU_0.97._

	N^1^	S^2^	ChaoI^3^	Shannon-Weiner diversity index, H	Evenness, H/H_max_ 4
Field population	89	6	NA	1.64	0.92
Seeds (symbiotic)	62	7	NA^6^	1.15	0.59
Seeds (aposymbiotic)	93	7	11.5	1.11	0.57
Prey (symbiotic)	87	9	9	1.76	0.80
Prey (aposymbiotic)	88	6	6.5	1.29	0.72
TOTALS	419	35			

1 Number of clones.

2 Observed number of OTU_0.97_ groups.

3 Chao1 = S + (n_1_)^2^/2n_2_ where n_1_ is the number of singletons and n_2_ is the number of doubletons.

4 Hmax  =  ln(S).

6 NA, not applicable (cannot be calculated because there were no doubletons).

Rarefaction curves (Fig. S1) and ChaoI estimates of species richness (Table 2) indicate that the libraries represented nearly all the species found in the Prey (symbiotic), Prey (aposymbiotic) and Field population treatments, but that libraries of the Seeds (symbiotic) and Seeds (aposymbiotic) treatments may have missed a few of the rarer community members. All data indicate that the carabid gut bacterial communities are simple, probably composed of less than 10 bacterial OTUs. By far, Gammaproteobacteria was the dominant bacterial class present in the clone libraries (57% of clones), followed by Mollicutes (13%), Cyanobacteria (10%), Bacilli (10%), Alphaproteobacteria (9%), and Betaproteobacteria (1%).

### Effect of treatment on bacterial community structure

**Figure 1:**
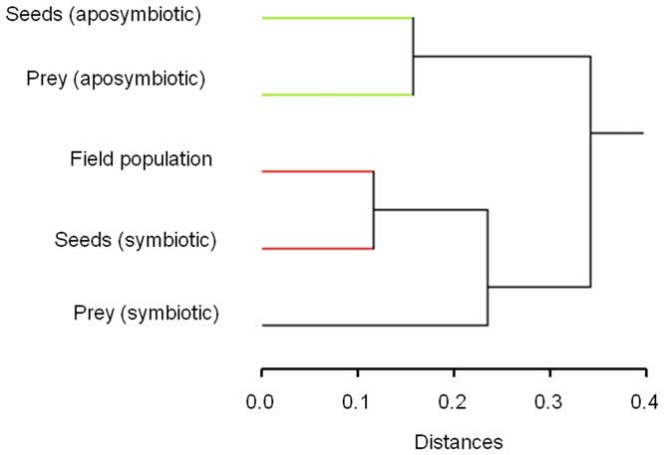
Relationships of bacterial communities in the beetles fed different diets. Cluster tree on the proportion of beetles in each dietary treatment that possessed each bacterial tRF. Tree distances are Euclidean, and a complete fusion strategy was employed for creating clusters. Branches of similar color are defined as clusters.

The dietary treatments were associated with different numbers of bacterial OTUs per beetle, and the relative abundances of each OTU varied among treatments. Based on the relative frequencies of individual bacterial tRF per treatment, the treatments grouped into two distinct clusters, one incorporating the two antibiotic-fed treatments, and one with the three treatments that were not exposed to antibiotics (Fig. 1). In the latter cluster, a sub-group with the shortest distance measured among all groups included the Field population and the Seed (symbiotic) treatments.

The analysis of the similarity in membership and relative abundance of tRF per beetle revealed that treatments varied significantly in their bacterial tRF profiles, except for the Field population and the Seed (symbiotic) treatment (Wilks' λ = 0.096, dfs = 18, 4, 70, *P*<0.001; α = 0.05). Mean ± SEM number of bacterial tRF per beetle were 3.10±0.48 (Field population), 3.06±0.51 (Seeds [symbiotic]), 1.15±0.32 (Seeds [aposymbiotic]), 4.88±0.81 (Prey [symbiotic]), 1.63±0.56 (Prey [aposymbiotic]). Eleven, four, four, one, and zero beetles in the Prey (aposymbiotic), Prey (symbiotic), Seeds (aposymbiotic), Seeds (symbiotic), and Field population treatments had no detectable bacteria.

**Figure 2:**
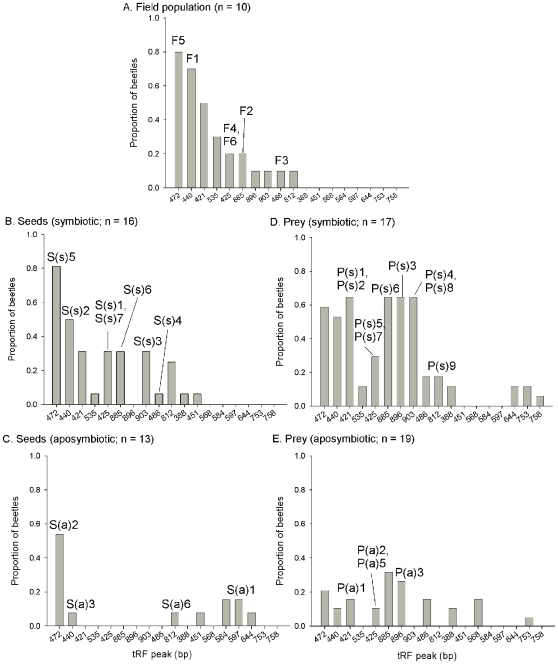
The proportion of each treatment that contained individual bacterial tRFs. Dietary treatments included A) Field population, B) Seeds (symbiotic), C) Seeds (aposymbiotic), D) Prey (symbiotic), and E) Prey (aposymbiotic). Numerical values in the sub-figure titles refer to the number of beetles analyzed. The arabic characters above each bar refer to samples in the clone libraries created for each treatment that have an identical tRF peak.

Eigenvalues for the two discriminant functions of use in describing the bacterial communities present in the different treatments were 1.45 and 1.00, and cumulatively described 43 and 73% of the dispersion in the datasets (Table 3). Treatment means of the canonical scores for each function are presented in Table 3, and revealed that the two functions described distinct treatment groupings; Function 1 described the strong differences in the canonical scores between the Seeds (aposymbiotic) and Prey (symbiotic) treatments, and Function 2 described the similarities between the Field population and the Seeds (symbiotic) treatments and their difference from the Prey (aposymbiotic) treatment. Function 1 is best described by the relative presences of tRF 421 (closest cultured matches from clone library with identical tRF: *Pantoea dispersa*), tRF 440 (*Ehrlichia shimanensis* or *Wolbachia pipientis*), tRF 472 (*Spiroplasma montanense*), tRF 896 (*Lactococcus garvieae* M79), and tRF 903 (*Enterococcus faecalis* RO90) (e.g, these bacterial tRFs had the five highest standardized canonical discriminant functions for Function 1). Function 2 is best described by the relative presences of tRF 421 (*Pantoea dispersa* UQ68J and *Enterobacter aerogenes*), tRF 472 (*Spiroplasma montanense*), tRF 885 (*Serratia fonticola*, *Seratia rubidaea*, *Seratia marcescens*, and *Acinetobacter calcoaceticus*), tRF 886 (*Lactococcus garvieae* M79), tRF 903 (*Enterococcus faecalis* RO90). These relationships are visualized in Figure 2.

**Table 3:** Canonical scores of group means and Eigenvalues for each discriminant function identified for the tRF-based bacterial communities per treatment (per beetle).

	Discriminant Function	
	1	2
Field population	−0.323	1.417
Seeds (symbiotic)	0.402	0.969
Seeds (aposymbiotic)	1.761	−0.060
Prey (symbiotic)	−1.819	−0.201
Prey (aposymbiotic)	0.254	−1.342
Eigenvalues (cumulative % of data dispersion)	1.45 (43%)	1.00 (73%)

### The influence of gut bacteria on food intake

**Figure 3:**
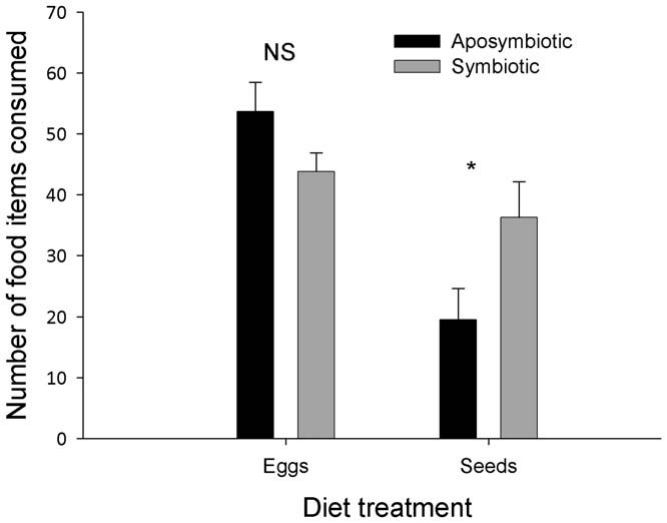
The effect of antibiotic treatment on mean (SEM) food consumption by *Harpalus pensylvanicus*. Beetles were fed prey (eggs of *Acheta domesticus*) or seeds (*Chenopodium album*) after being treated with a dietary source of antibiotics for 10 d. An asterisk indicates significant differences between log-transformed means within a food category (α = 0.05).

There was no effect of antibiotics on *H. pensylvanicus*' consumption of prey (F_1, 34_ = 1.27, *P* = 0.27), but consumption of antibiotics was associated with a 40% reduction in seed consumption (F_1, 26_ = 7.29, *P* = 0.01; Fig. 3). The reduction in seed consumption was only observed in males (mean ± SEM seed consumption: symbiotic ♂♂, 39.7±8.55 [n = 10]; aposymbiotic ♂♂, 18.29±4.69 [n = 7]; F_1, 15_ = 4.03, *P* = 0.06), but not in females (symbiotic ♀♀, 24.83±5.79 [n = 6]; aposymbiotic ♀♀, 21.00±10.33 [n = 6]; F_1, 10_ = 0.43, *P* = 0.52). A significant stepwise GLM was created to describe the relationship between bacterial presence/absence and seed consumption in the symbiotic beetles (regression: F_2, 13_ = 15.04; *P*<0.001; r^2^ = 0.70). Only two bacterial tRFs (535 & 903) in the symbiotic treatment were statistically correlated with seed consumption to be included in the stepwise GLM, those beetles with 903 were positively and those with 535 were negatively associated with seed consumption (constant: t = 18.13, *P*<0.001; 535: t = −4.26, *P* = 0.001; 903: t = 2.66, *P* = 0.02). tRF 903 corresponds to the cloned bacterial sequence which most closely matches *Enterococcus faecalis* R090 (Table 1), which was totally removed from populations fed antibiotics (Fig. 2). Beetles that possessed *E. faecalis* consumed a mean (SEM) of 56.40±12.41 seeds per beetle, and those without *E. faecalis* consumed 22.63±3.47 seeds. This bacterial tRF was found in 64.71% of Prey-fed (symbiotic) beetles, 31.25% of Seed-fed (symbiotic) beetles, and 10% of the Field population. Only males in the Seed-fed (symbiotic) and Field populations harbored *E. faecalis*, whereas eight of 11 beetles in the Prey-fed (symbiotic) that had *E. faecalis* were females. Only one symbiotic beetle was found to possess tRF 535, and this insect only consumed two seeds. This tRF was not identified in the clone libraries, and occurred exclusively in the symbiotic treatments (including the Field treatment) (Fig. 2).

## Discussion

This research confirms that a bacterial community present in the guts of a facultatively granivorous beetle is associated with its ability to consume seeds. Consuming different foods alters this bacterial community, and antibiotic treatment reduces overall bacterial populations and the relative abundance of specific bacterial taxa without directly influencing the host insect. Finally, we suggest a putative function for one of the bacteria: *Enterococcus faecalis* may be a symbiont that facilitates granivory in this omnivorous beetle. The result is an underscoring of the importance of a facultative nutritional symbiosis as a mechanism for explaining dietary breadth in this group of beneficial insects.

### Bacterial community in the guts of an omnivorous beetle

The current research confirms previous assessments that carabid beetles mirror many other insects by possessing taxonomically simple bacterial communities within their guts. The clone libraries identified 25 bacterial OTUs in our entire population of 75 beetles (Table 1), and the tRF analysis revealed 18 distinct peaks (Fig. 2). It is important to note that a known weakness of tRF analysis is that multiple bacterial taxa may express a single tRF peak, which explains why different OTUs identified in the clone libraries produced identical tRF peaks. Most of the clones were indicative of bacterial groups known to reside symbiotically with animals and plants (i.e., Gammaproteobacteria & Alphaproteobacteria). Previous research showed that the bacterial gut communities of Collembola [57], Coleoptera [58], [59], Diptera [60], Heteroptera [6], [7], [61], Hymenoptera [62], [63], Lepidoptera [64], [65], and Neuroptera [66] are equally if not more simple than those of *H. pensylvanicus*. Also similar to our results, these previous studies isolated primarily those bacterial taxa known for symbiotic associations with animals and plants [67], [68], rather than those groups commonly isolated from the soil or other sources. Sometimes these gut symbionts of insects pervade throughout an insect population, especially when the insect has physiological adaptations in their digestive systems that house bacterial symbionts (e.g., gastric caecae or structurally complex alimentary canals) [1], [61], [69], [70], [71]. Only a few of the bacteria were found in more than 50% of the symbiotic *H. pensylvanicus* population, notably *Spiroplasma montanense* (tRF 472; Mollicutes), Alphaproteobacteria (tRF 440, closest genetic matches were *Wolbachia pipientis* and *Ehrlichia shimanesis*, whose genetic similarities to the clones were approximately 87%), and a Gammaproteobacteria (tRF 421; closest genetic matches were *Pantoea dispersa* UQ68J and *Enterobacter aerogenes*, whose genetic similarities to the clones were between 96.2–97.4%) (Table 1, Fig. 2). The majority of the bacterial community was much less pervasive (fewer than 50% of the beetles), and apparently strongly influenced by the intrinsic and extrinsic conditions associated with their hosts.

### The effect of diet and antibiotics on gut bacterial communities

An animal's diet often influences which bacteria reside within its gut and vice versa. In our study, the gut communities of field populations of *H. pensylvanicus* were most similar to the lab populations fed seeds (symbiotic) (Fig. 1), which may be indicative of the facultatively granivorous lifestyle of this species observed in natural conditions [72], [73], [74]. These two populations shared all but a minor three of their tRF peaks and had similar relative abundances of their predominant peaks, whereas the prey fed (symbiotic) treatment differed from the field population in the presence of four peaks, and the relative abundances of 885 (*Serratia* spp.), 896 (*Lactococcus garviae*), and 903 (*Enterococcus faecalis*) were found in substantially more beetles in the prey (symbiotic) treatment than in the Field population (Fig. 2). *Lactococcus garvaeae* was also found in the stomachs of the more predatory carabid, *Poecilus chalcites* [44] and the stomachs of fire ants (*Solenopsis invicta*) [62]. Other research has found that changes in an insect's diet accompany changes in bacterial gut communities [64], [75]; for example, substantially different bacterial communities resided within cohorts of *Lymantria dispar* (Lepidoptera: Lymantriidae) caterpillars fed different host plants [64]. Although diet-associated changes in bacterial symbionts are well documented [4], [8], [76], the implications remain poorly understood for gut-based bacterial communities, but see [59]. One possible function is that these transient, food-associated bacterial species may possess the means to digest the food substance, a trait which can be harnessed by the host insect [77].

Not surprisingly, antibiotic treatment reduced the overall abundance of bacteria, and changed the species of bacteria found within the guts of *H. pensylvanicus* (Table 1, Figs. 1 & 2) [65]. In both prey- and seed-fed treatments, antibiotics reduced the number of tRF peaks per beetle by approximately 60–70% (to a mean less than 1.63 tRF per beetle), and those tRF peaks found in the symbiotic treatments were invariably less abundant in the aposymbiotic treatments. Moreover, new tRF peaks were isolated from antibiotic-fed insects that were not found in the symbiotic treatments. One such peak (597) was identified in the clone libraries (clone S[a]1; Table 1) as most similar (98%) to an uncultured Cyanobacteria recovered from throat aspirates of humans receiving antibiotics. Indeed, in the seed fed treatment, antibiotics clearly shifted the community away from Gammaproteobacteria (only 1% of clones in the aposymbiotic treatment were Gammaproteobacteria, versus 77% in the symbiotic treatment) and toward Cyanobacteria and Mollicutes (Table 1). This same taxonomic shift was not observed in the prey-fed treatments (Table 1), and may reflect that the beetles receive components of their gut fauna from their diet. Also noteworthy is that antibiotic treatment entirely removed the bacterium, *E. faecalis* from the beetle population, an effect to be discussed more below. The result is that the bacterial communities within aposymbiotic treatments were more similar to each other than to any of the other treatments (determined with cluster analysis), regardless of what food they consumed (Fig. 1).

### The effect of treatment on seed consumption

Beetles fed antibiotics ate fewer seeds than untreated beetles, and this effect was extraordinarily strong for beetles that harbored *E. faecalis* in their guts. Beetles ate similar numbers of cricket eggs whether they were treated with antibiotics or not (Fig. 3), indicating that antibiotic treatment did not have noticeable direct physiological effects on the beetles, or alter their feeding behavior when provided with prey. In contrast, *H. pensylvanicus* fed antibiotics ate 43% fewer seeds on average (Fig. 3). This treatment effect was driven by only seven of the 13 antibiotic-treated beetles (six of the beetles receiving antibiotics ate more than 25 seeds, similar to the symbiotic treatment). Three of the 16 untreated beetles ate fewer than 10 seeds. A closer examination of the bacterial community present in each of these beetles found that the presence of only one bacterial OTU was consistently correlated with high levels of seed consumption, *E. faecalis*. This bacterium has been isolated from the guts of other herbivorous insects [64], [78], [79], [80], and the strain of closest genetic similarity (R090) to ours was isolated from fermenting rice silage in Asia [81]. Broderick et al [64] postulated that *Lymantria dispar* caterpillars fed antibiotics became more susceptible to the entomopthogen, *Bacillus thuringiensis*, possibly because the common gut resident, *E. faecalis*, acidifies the gut environment. Under some conditions, some biotypes of *E. faecalis* are believed to be pathogenic to insect hosts [82], [83]. Although this bacterium was the most commonly found bacterium in cadavers of two stalk-boring caterpillars (*Diatraea* spp.) across four study locations, only 22% of caterpillars inoculated with this bacterium died [78]. We add possible contributions to seed digestion in facultatively granivorous beetles to the list of putative roles of *E. faecalis.*

Strains of *E. faecalis* are often considered to be opportunistic pathogens of clinical significance commonly living a commensal existence in the guts of warm-blooded animals. High abundances of Enterocci, often *E. faecalis*, and their possible role in insect diseases are commonly documented in the literature [84]. However, a chief finding of Martin and Mundt [84] was that the strains of *E. faecalis* recovered from insects were physiologically distinct from those recovered from clinical specimens, suggesting additional roles for this organism in symbiotic relationships. More recent studies have shown that *E. faecalis* and other Enterococci were prominent within bark beetles [85], houseflies [86], fruitflies [87]; grasshoppers and locusts [88], gypsy moth larvae [64], wood termites [89] and were the most active bacterium within *Manduca sexta* [90]. Functional roles postulated for *E. faecalis* in insects range from vectoring antibiotic resistance genes [86], modulating parasite transmission [91], to nutritional upgrading [85], [90]. *E. faecalis* is usually considered a homofermentative organism producing lactic acid by fermenting cellulosic sugars, a function that is exploited in some settings, e.g. silage production [81]. It may be expected that this function contributes to the dietary needs of *H. pensylvanicus*. A related Enterococci strain is thought to produce acetic acid, instead of lactic acid, in the microaerophilic environment of the termite hindgut [89].

In summation, the functions of facultative symbionts in the guts of animals remain poorly understood, but it appears that even loose associations of individual hosts with specific bacteria can result in dramatically different host phenotypes. Regardless of whether diet affects the bacterial community or the bacterial community affects the hosts' diet, the end result is that very different diets can arise sympatrically within an animal population, depending on the bacterial symbiotic relationships that occur. This study underscores the notion that the nutritional ecology of an organism can only be understood in the context of the host and its microbial symbionts, and even bacteria that are not obligate symbionts can have important implications for the dietary breadth of an animal species.

## Supporting Information

Figure S1 Rarefaction analysis of the bacterial 16S rRNA gene clone libraries from the five groups of beetles.
(9.57 MB TIF)
